# Supplementary material for: Chromothripsis during telomere crisis is independent of NHEJ, and consistent with a replicative origin
Source: Genome Res. 2019 May;29(5):737–49. doi: 10.1101/gr.240705.118 (PMC6499312; doi:10.1101/gr.240705.118)
Supplement: Supplemental Material [file supp_gr.240705.118_Supplemental_file_1.zip › contigs/annotated_contigs/DB88/contig.2.DB88_length_466_mean_cov_12.2317596567.docx]

**DB88_length_466_mean_cov_12.2317596567**

```
GCAGCAGATGGCTCACAAAGAATGGGTAGGGAAAGTGTCTATACCATCAGTCTCATGGAAAGGATCCTTGCTTGGTACCCACTCTTCAG
 >chrX:17613233-17613439 - E=2e-112
GATCAAAGAATGAGCTTGCAGGTGAGCTATCTGCACCCAGCAAGGCAGCCTGCATCTGAATGAGAAGAGCCAAAATCCCTACAGAACTT

CTACATATATTCCATGTCAGCAGGAACA|GGGCAGGAACAGGAACAGCCCTGTTCCTGCTGTGATTTCATTCAACAGGAACA|GGGCTC
                                                                                  >chr1
TCTGTGTATCTAAGAGCTCATGTCTGGGATGAGTGAGTTCTGGTATCTGGGCCTGCACTTCTCACCACTGGCCCTGACTCCTCCCTTAG
4:107219502-107219709 + E=5e-113
CCAACTCCAGGACACAGCCGGCCAGGCCTAGTGTGGTTTGCAGAAGCCACTGCCTAATCAGAATATGGATGATTTTCCTGCACTCTCCT

GTTTACCTTAAACTATGGAGAGA
```
